# Atmospheric Carbonyl Compounds at Shangdianzi, Beijing: Autumn-to-Winter Variation, Ozone Formation Potential, and Source Apportionment

**DOI:** 10.3390/toxics14020156

**Published:** 2026-02-04

**Authors:** Yufei Song, Xiaoshuai Gao, Junling Li, Shudan Wei, Yushi Gong, Haijie Zhang, Yanqin Ren, Yucong Guo, Weigang Wang, Hong Li, Maofa Ge

**Affiliations:** 1 State Key Laboratory of Environmental Criteria and Risk Assessment, Chinese Research Academy of Environmental Sciences, Beijing 100012, China; 2 Institute of Chemistry, Chinese Academy of Sciences, Beijing 100190, China

**Keywords:** carbonyl compounds, pollution characteristics, ozone formation potential (OFP), source analysis, Shangdianzi

## Abstract

Based on continuous field observations conducted at the Shangdianzi Regional Atmospheric Background Station from 21 October to 20 November 2024 and from 1 December 2024, to 2 January 2025, this study systematically analyzed the concentration levels, seasonal variations, diurnal patterns, and ozone formation potential (OFP) of 24 carbonyl compounds (OVOCs) in the atmosphere during autumn and winter. Source apportionment was further investigated using characteristic ratios, correlation analysis, and multiple linear regression. The results indicate that the average concentration of Σ24OVOCs during the observation period was 2.70 ± 1.55 ppb. Formaldehyde, acetone, and acetaldehyde were the dominant species, accounting for 94.5% of the total concentration in this background area. A significant seasonal difference in carbonyl concentrations was observed, with the average concentration in autumn (3.68 ± 1.66 ppb) being approximately 2.1 times higher than that in winter (1.78 ± 0.58 ppb). The diurnal variation in most carbonyls exhibited a pattern of nighttime accumulation and daytime depletion, which was consistent with the trend of NO_2_. The OFP results show that the average OFP of Σ24OVOCs was 30 ± 16 μg/m^3^, with formaldehyde contributing 86.9%, identifying it as a key precursor for ozone formation in the background region. Source analysis revealed that carbonyl compounds in autumn were influenced by combined natural, vehicular, and industrial sources, with significant secondary formation (27–36%) observed for C2 (acetaldehyde) and C3 (mainly acetone and propanal) species. In winter, anthropogenic contributions to carbonyls increased, with C2 and C3 species primarily originating from combustion sources, vehicle emissions, and industrial releases. This study provides the first insights into the pollution characteristics and source profiles of carbonyl compounds during autumn and winter at the Shangdianzi background site, offering a scientific basis for understanding regional atmospheric oxidative capacity and formulating integrated air pollution control strategies.

## 1. Introduction

Carbonyl compounds are important oxygenated volatile organic compounds (OVOCs) in the atmosphere, playing a critical role in tropospheric photochemical processes [1,2]. They serve as significant sources of various free radicals (e.g., OH, HO_2_, and RO_2_) and participate in the formation of ozone (O_3_), peroxyacetyl nitrate (PAN), and secondary organic aerosols (SOA), thereby directly influencing regional atmospheric oxidative capacity [3,4]. The atmospheric photochemistry of carbonyl compounds and their connections to alkene oxidation have been widely studied since the 1980s and 1990s. Early work established carbonyls as key intermediates and products in alkene oxidation and highlighted the importance of carbonyl photolysis in regulating radical chemistry and ozone formation in the troposphere. Seminal reviews by Atkinson and co-workers synthesized reaction mechanisms and kinetic data for carbonyl and alkene chemistry, providing a foundational framework for subsequent atmospheric studies and the present investigation [5,6,7,8,9]. Meanwhile, most carbonyl compounds pose significant health risks and have been classified as toxic air pollutants by the World Health Organization. Formaldehyde, in particular, has been identified as a definite carcinogen [10,11], while others such as acetone and acetaldehyde are associated with respiratory and hematologic toxicity [10,12,13]. Therefore, identifying the ambient concentration characteristics and sources of carbonyl compounds is of great significance for deepening the understanding of regional photochemical processes and atmospheric complex pollution.

Carbonyl compounds in the troposphere originate from complex sources, including direct primary emissions [14,15] and secondary formation through the oxidation or photolysis of VOCs [16]. Primary sources can be further classified into natural and anthropogenic origins. Natural sources primarily involve plant volatilization and biomass burning, while anthropogenic sources encompass vehicle emissions, industrial processes, solvent use, and cooking oil fume release, among others [17,18,19,20,21]. In recent years, observational studies on carbonyl compounds have been conducted in numerous Chinese cities, revealing their distinct diurnal and seasonal variations and indicating that their concentrations are jointly influenced by temperature, solar radiation, and anthropogenic emission intensity [22,23,24,25,26]. Generally, the concentrations of carbonyl compounds in urban areas are higher than those in suburban and rural areas within the same region, while suburban concentrations are more sensitive to regional transport [27].

Atmospheric carbonyl compounds, due to their high reactivity and susceptibility to oxidation, impose relatively high technical requirements for their observation. Derivative-based methods are commonly employed for their measurement. In recent years, extensive research in this field has been conducted by scholars both domestically and internationally, primarily focusing on pollution characteristics, major sources, key component reactivity, and their contributions to ozone formation. Within China, observational studies on carbonyl compounds have been predominantly concentrated in urban areas of the Beijing-Tianjin-Hebei region, the Pearl River Delta, the Yangtze River Delta, and their surrounding regions [22,23,24,25,26]. Internationally, countries such as France [28], Finland [29], Italy [30], Brazil [31], and the United States [32,33,34,35] have also conducted extensive observational studies. Compared to urban areas in other countries, carbonyl compound concentrations in Chinese cities remain at relatively high levels. Despite the increasing volume of related research, most studies focus on urban environments or pollution episodes, leaving long-term continuous observations of carbonyl compounds in atmospheric background areas relatively scarce.

As a key atmospheric background station in North China, the Shangdianzi station is remote from major pollution sources and serves to represent regional-scale background atmospheric conditions [36,37,38]. Its unique geographical location not only allows it to characterize the background features of northern air masses under the influence of the East Asian monsoon system but also provides a natural advantage for monitoring cross-regional transport processes, such as pollution influx from the Mongolian Plateau, Northeast China, and outflow from the Beijing-Tianjin-Hebei region. However, systematic research on the concentration levels, compositional profiles, and sources of carbonyl compounds in the North China background region remains insufficient. This knowledge gap hinders the understanding of photochemical processes in background areas and their interactions with urban pollution. Furthermore, quantitative analyses of the role of carbonyl compounds in ozone formation potential (OFP) and their seasonal variations in background regions are still lacking. Therefore, this study conducted continuous observations at the Shangdianzi background station during the autumn and winter of 2024. It systematically analyzed the concentration levels, compositional characteristics, seasonal variations, and diurnal patterns of 24 carbonyl compounds. The maximum incremental reactivity (MIR) method was employed to assess their OFP. Additionally, source apportionment was performed using characteristic ratios, correlation analysis, and multiple linear regression. The findings aim to fill the gap in multi-species carbonyl observations in the North China background region and provide a scientific basis for a deeper understanding of regional atmospheric oxidative capacity and for formulating coordinated control strategies for ozone and VOCs.

## 2. Materials and Methods

### 2.1. Sample Collection

**Figure 1:**
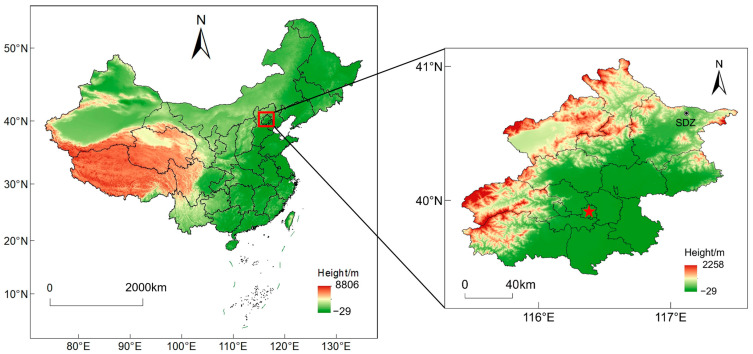
Geographic location of the Shangdianzi background monitoring station (the red five-pointed star in the figure indicates the location of Beijing’s main urban area).

This study selected the Shangdianzi Atmospheric Background Monitoring Station (40.65° N, 117.12° E, height above mean sea level: 293.9 m) as the site for carbonyl compound observations. The station is located in Shangdianzi Village, Gaoling Town, Miyun District, Beijing, with an approximate straight-line distance of 100 km from downtown Beijing. As one of the atmospheric background stations established in China under the World Meteorological Organization Global Atmosphere Watch (WMO/GAW) program, it is among the earliest nationally recognized field scientific observation and research stations dedicated to obtaining atmospheric environmental background values in China. Figure 1 shows the specific location of the station. The station’s geographical location is remote from urban built-up areas and population centers, with no other significant pollution emission sources in the vicinity.

Sampling was conducted during autumn and winter, covering the periods from 21 October to 20 November 2024 and from 1 December 2024 to 2 January 2025, respectively. A semi-automatic carbonyl sampler was employed for continuous collection of atmospheric carbonyl compounds using 2,4-dinitrophenylhydrazine (DNPH) [39]. Sampling was performed daily across six time intervals: 00:00–04:00, 04:00–08:00, 08:00–12:00, 12:00–16:00, 16:00–20:00, and 20:00–00:00, with a flow rate of 0.5 L·min^−1^. Field blank samples were collected at the beginning, middle, and end of the sampling campaign. The blank sample was prepared by installing a sampling cartridge at the sampling location without activating the sampling pump, leaving it in place for the same duration as the actively sampled cartridges, and then retrieving it for analysis. These blank samples were stored and analyzed under the same conditions as the environmental samples to allow for background subtraction during final data processing to account for potential contamination introduced during sampling and analysis. To prevent O_3_ from reacting with the formed hydrazone derivatives and affecting the results, a potassium iodide (KI) (Tianjin ChengZhou TUPlabs Technologies Co., Ltd., Tanjing, China) scrubber was installed upstream of the DNPH cartridge and the sampler inlet to remove ambient O_3_ before it could interact with DNPH and the generated hydrazone compounds [40].

The sampler utilizes a mass flow controller (MFC) to regulate the gas flow rate. Prior to the start of each sampling phase, the flow rate of the MFC was calibrated using a flow calibrator (520 M, Mesalabs, Lakewood, CO, USA). The sampler flow rate was checked and calibrated weekly to ensure its accuracy, with the actual sampling flow rate recorded as the value displayed by the flow calibrator. The semi-automatic carbonyl sampler can collect up to six samples per cycle. During the sampling campaign, sampling cartridges were replaced daily at 16:00. After cartridge replacement was completed, both ends of the sampling cartridge were sealed, wrapped in aluminum foil, and placed in a cold storage box. Since the analytical process in this study spanned several months, the cartridges were stored at −18 °C to maximally inhibit any potential degradation or loss of the derivatives, particularly those of highly reactive aldehydes. A total of 384 samples were collected in this study, comprising 186 samples from autumn and 198 samples from winter.

During the sampling period, data on wind direction, wind speed, temperature, humidity, PM_2.5_, and other parameters were monitored in real-time by the automatic weather station at the observation site. The photolysis rate of NO_2_ (JNO_2_) was measured by a NO_2_ photolysis rate constant instrument (METCON, North Rhine-Westphalia, Germany). Data for O_3_, SO_2_, NO, and NO_2_ were obtained from an O_3_ analyzer (Thermo 49i, Waltham, MA, USA), an SO_2_ analyzer (Thermo 43i, Waltham, MA, USA), and a NOx analyzer (Thermo 42i, Waltham, MA, USA), respectively. CO data were acquired using a CO gas analyzer (Picarro G2401, Santa Clara, CA, USA).

### 2.2. Sample Analysis

Atmospheric carbonyl compounds reacted with DNPH coated on the sampling cartridges to form hydrazone derivatives. These derivatives were then slowly eluted from the cartridge using acetonitrile and diluted to a final volume of 5 mL. The eluate was then transferred into 1.5 mL amber high-performance liquid chromatography (HPLC) vials. To ensure the stability of some highly reactive derivatives over the extended time frame and to prevent evaporation of the acetonitrile solvent, the vials were stored at −18 °C pending instrumental analysis. For quantification, a carbonyl-DNPH derivative mixed standard was used. This standard was diluted with acetonitrile to create a series of concentrations for establishing calibration curves for the target carbonyl compounds. However, during the subsequent analysis, butyraldehyde, isobutyraldehyde, and 2-butanone could not be chromatographically separated and were therefore quantified collectively as 2-butanone. Consequently, the number of carbonyl species quantified in this study was effectively 24. Specific analytical techniques have been described elsewhere [39].

The analytical instrument employed in this study was an HPLC system (LC-20AD, Shimadzu, Kyoto, Japan) equipped with an ultraviolet detector (SPD-20A, Shimadzu, Japan). The analytical column was a 250 mm × 4.6 mm, 5 μm C18 column (Inertsil ODS-SP, Shimadzu, Japan). Throughout the analysis period, the guard column (Inertsil ODS-SP 5 μm, Shimadzu, Japan) was replaced promptly based on changes in column pressure. The replaced guard columns were cleaned ultrasonically for subsequent reuse. Prior to aspirating sample solutions, the autosampler of the HPLC system was purged with a 50% (*v*/*v*) methanol-water mixture. The system was coupled in series with a triple quadrupole mass spectrometer (API3200, AB Sciex, Framingham, MA, USA) for tandem mass spectrometry (MS/MS). Acetonitrile and deionized water were used throughout the sample analysis process.

The liquid chromatography system automatically injected 20 μL of the prepared sample solution from the HPLC vial into the HPLC-MS/MS system via the autosampler. The analysis employed a binary gradient elution with acetonitrile (A) and water (B). The gradient program was as follows: 60% A was held for 20 min; from 20 to 30 min, A was linearly increased from 60% to 100%; from 30 to 32 min, A was decreased back to 60%; this composition was then maintained for an additional 8 min, resulting in a total analysis time of 40 min per sample. The column was housed in a thermostatted compartment maintained at a constant temperature of 40 °C. For mass spectrometric analysis, an atmospheric pressure chemical ionization (APCI) source in negative ion mode was used. The ion source temperature was set to 400 °C with a curtain gas flow of 50 psi. Data acquisition was performed using the Q1 Multiple Ions mode. Concentration data for the 24 target carbonyl compounds were obtained and processed using the AB SCIEX Analyst software (https://sciex.com.cn/products/software) (accessed on 3 January 2025). Prior to routine analysis, detection performance was evaluated under various ion source heating temperatures and gas flow rates to establish the optimal conditions yielding the highest response. Furthermore, the chromatograms for each compound were individually inspected to ensure the accuracy of peak area integration.

### 2.3. Quality Assurance and Quality Control (QA/QC)

The concentrations of carbonyl compounds were quantified using the external standard method. A mixed stock standard solution of the 24 target carbonyl-DNPH derivatives at a concentration of 15 μg/mL was serially diluted to prepare calibration solutions at different concentration levels (0.6, 1.2, 2.4, 3.6, 6, 12, 18, 30, 60, 120, 180, 300, and 600 ng/mL). Calibration curves were constructed using these mixed standard solutions. The linear range for all 24 carbonyl compounds was 0.6–600 ng/mL. Information on the DNPH-derivatized carbonyl standards, mass spectrometry (MS) acquisition parameters, method detection limits (MDLs), and correlation coefficients is presented in Table 1. The correlation coefficients for most compounds were greater than 0.995. The MDLs ranged from 0.2 to 0.9 ng/mL. The relative standard deviation (RSD) for triplicate injections on the same day was below 4.2%, and the RSD for analyses conducted on three consecutive days was below 12.5%. To validate the sampling efficiency, two DNPH cartridges were connected in series and sampled simultaneously for a specific period. The carbonyl concentrations in both cartridges were subsequently analyzed. The collection efficiency for formaldehyde, acetaldehyde, and acetone was found to exceed 99% at the sampling flow rate of 0.5 L/min. Field blank samples were collected and analyzed at the beginning, middle, and end of the sampling campaign. The results showed that the concentrations of the target compounds in all blank samples were below their respective method detection limits.

### 2.4. Ozone Formation Potential (OFP)

OFP was calculated using the maximum incremental reactivity (MIR) of VOCs to assess the contribution of different carbonyl compounds to O_3_ formation. The OFP calculation formula is shown in Equation (1):

(1) $${OFP}_{i}={MIR}_{i}\times \left[{VOC}_{i}\right]$$

In the equation, OFP_i_ represents the maximum ozone formation potential of VOC species i (μg/m^3^); MIR_i_ denotes the maximum incremental reactivity of VOC species i, which is the maximum O_3_ concentration produced per unit mass of VOC added (g O_3_/g VOC); and [VOC_i_] is the mass concentration of VOC species i (μg/m^3^). The MIR values for individual VOCs are adopted from references [1,41].

### 2.5. Multiple Linear Regression

The concentration of carbonyl compounds from various sources shows significant correlations with tracer species representative of typical pollution sources. Based on this characteristic, it is assumed that there is a linear relationship between the primary and secondary sources of carbonyl compounds and the selected tracers. Therefore, different pollutants can be selected as characteristic tracers for primary emissions and secondary pollution (e.g., CO and O_3_) and incorporated into a multiple linear regression model for simulation [42,43,44,45,46]. This approach allows for the distinction between primary emissions and secondary formation contributions to carbonyl concentrations. Notably, discrimination of the background sources (such as natural Sources and long-term accumulation in the atmosphere) could not be achieved by the statistical analysis [44,45,46,47]. The specific calculation formula for multiple linear regression is as follows:

(2) $$\left[Carbonyl\right]=\beta_{0}+\beta_{1}\left[{Tracer}_{1}\right]+\beta_{2}\left[{Tracer}_{2}\right]$$

In the equation, [Carbonyl], [Tracer_1_], and [Tracer_2_] represent the concentrations of the target carbonyl compound, the primary emission tracer, and the secondary formation tracer, respectively; β_0_ represents the background concentration of the target carbonyl compound; β_1_ and β_2_ are the coefficients corresponding to primary emission and secondary formation contributions, respectively. The contribution calculations for primary emission, secondary formation, and background sources are as follows:

(3) $${Primary}_{i}=\frac{{\beta_{1}\left[{Tracer}_{1}\right]}_{i}}{{\left[Carbonyl\right]}_{i}}\times 100\%$$

(4) $${Secondary}_{i}=\frac{{\beta_{2}\left[{Tracer}_{2}\right]}_{i}}{{\left[Carbonyl\right]}_{i}}\times 100\%$$

(5) $${Background}_{i}=\frac{{\beta_{0}}_{i}}{{\left[Carbonyl\right]}_{i}}\times 100\%$$

In the equation, Primary_i_, Secondary_i_, and Background_i_ represent the contributions from primary emissions, secondary formation, and background sources, respectively, for the target carbonyl compound i; [Carbonyl]_i_ denotes the concentration of the target carbonyl compound i; [Tracer_1_]_i_ and [Tracer_2_]_i_ represent the concentrations of the corresponding primary emission tracer and secondary formation tracer, respectively, for the target carbonyl compound i.

## 3. Results and Discussion

### 3.1. Air Quality and Meteorological Conditions During the Observation Period

During the observation period, the air quality monitored at the Miyun Town station (40.37° N, 116.83° E, height above mean sea level: 82 m), located near the Shangdianzi background station, remained generally good, with no severe pollution episodes recorded [48]. However, significant seasonal differences in air quality were observed. The average Air Quality Index (AQI) value in autumn was 66.9, which overall falls within the “Good” category. While short-duration, slight pollution episodes possibly influenced by regional transport or local accumulation occurred during this season, the overall air quality was satisfactory. In winter, the average AQI value decreased to 33.7, reaching the “Excellent” category, indicating air quality was significantly better than in autumn. The AQI for both seasons remained below 100, consistent with the characteristics of clean air at a regional background station. This seasonal variation is likely closely related to differences in meteorological conditions, changes in emission sources, and the seasonal characteristics of atmospheric chemical processes.

**Figure 2:**
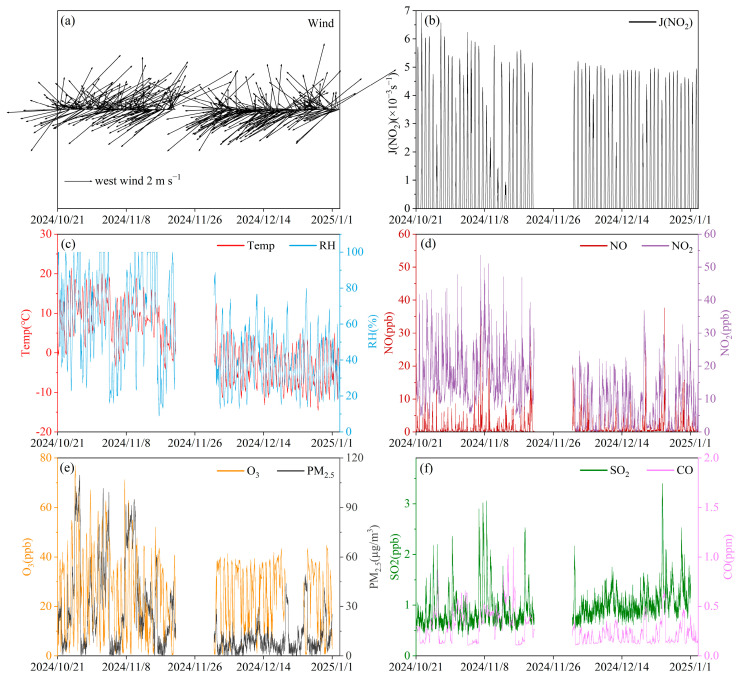
Time series variations during the sampling period at the Shangdianzi background station: (**a**) wind direction and speed, (**b**) JNO_2_, (**c**) temperature and relative humidity, (**d**) NO and NO_2_, (**e**) O_3_ and PM_2.5_, and (**f**) SO_2_ and CO.

Significant seasonal differences in meteorological conditions were observed at the Shangdianzi background station from October 2024 to January 2025 (Figure 2). The average temperature in autumn was markedly higher than in winter, fluctuating between 1.0 and 13.5 °C, whereas winter temperatures dropped to a range of −7.1 to 2.9 °C. The higher autumn temperatures, combined with stronger solar radiation (daily average JNO_2_ range: (0.5–4.0) × 10^−3^ s^−1^), promoted regional photochemical reactions. Conversely, weaker radiation in winter ((0.7–3.2) × 10^−3^ s^−1^) limited photochemical activity. Relative humidity also showed distinct seasonal variation. Autumn humidity was generally higher and more variable, with maxima reaching up to 100% influenced by precipitation events, while winter humidity was overall lower (28.4–58.7%), reflecting differences in water vapor conditions and cloud cover. Wind speeds remained low during both seasons (autumn: 1.1–3.7 m/s; winter: 1.4–3.4 m/s, The specific wind direction, wind speed, and wind rose diagrams are shown in Figure 2 and Figure S1), indicating that the Shangdianzi area was characterized by weak atmospheric dispersion, a condition conducive to the nighttime accumulation of pollutants [36,37,38].

Regarding conventional pollutants, both O_3_ and NO_2_ exhibited typical photochemical formation and depletion characteristics. The daily average O_3_ concentration ranged from 8.6 to 40.6 ppb in autumn and 12.9 to 35.5 ppb in winter. Although the overall levels were low, distinct diurnal variation patterns were maintained. NO_2_ showed a “high at night, low during the day” pattern in both seasons, which was highly consistent with the diurnal variation of carbonyls discussed later. Concentrations of PM_2.5_, SO_2_, and CO remained at low levels typical of background sites. Among them, the stable variation pattern of CO made it a suitable tracer for primary emissions in the subsequent multiple linear regression analysis.

Overall, the Shangdianzi station had stronger conditions conducive to photochemical reactions in autumn, while atmospheric oxidative capacity decreased significantly in winter. These meteorological differences established a crucial background context for the seasonal and diurnal variations of carbonyl compounds, directly influencing their secondary formation, photolytic loss, and the accumulation and dispersion processes governed by boundary layer dynamics.

### 3.2. Concentration Levels and Variation Characteristics of Carbonyl Compounds

#### 3.2.1. Overall Concentration Levels and Compositional Characteristics

**Figure 3:**
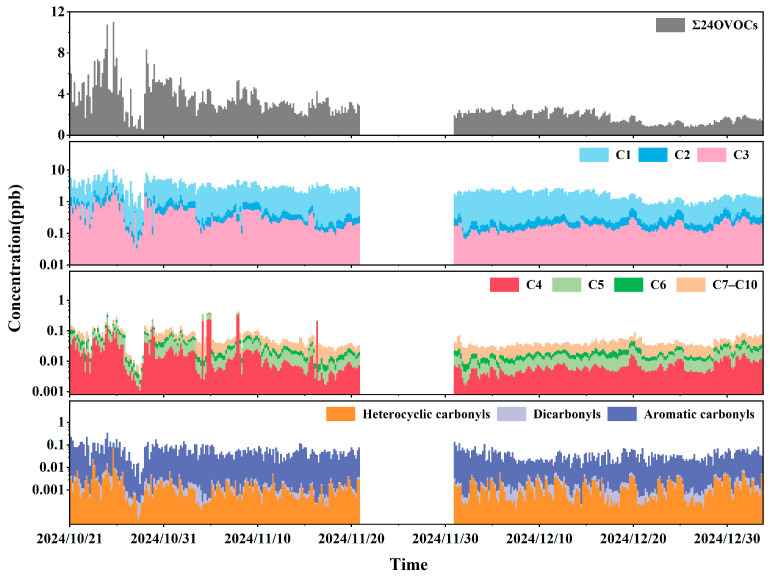
Time series of atmospheric carbonyl compounds during the sampling period at the Shangdianzi background station. (C1: Formaldehyde; C2: Acetaldehyde; C3: Acetone + Propanal + Acrolein; C4: Crotonaldehyde + Methacrolein + 2-Butanone; C5: n-Valeraldehyde + Isovaleraldehyde; C6: n-Hexanal + Cyclohexanone + Methyl isobutyl ketone; C7–C10: n-Heptanal + n-Octanal + n-Nonanal + n-Decanal; Heterocyclic Carbonyls: 2-Furaldehyde; Dicarbonyls: Glutaraldehyde; Aromatic Carbonyls: Benzaldehyde + o-Tolualdehyde + m-Tolualdehyde + p-Tolualdehyde + 2,5-Dimethylbenzaldehyde. The sampling duration for each sample was 4 h.).

The time series of carbonyl compound concentrations during the observation period is shown in Figure 3, revealing significant phased fluctuations in both autumn and winter. Since the observations began on 21 October, the total concentration of the 24 carbonyl compounds (Σ24OVOCs) generally exhibited a trend of initial increase followed by a decrease. The highest daily average Σ24OVOCs concentration of 6.9 ppb was recorded on 25 October, with the peak four-hour average concentration reaching 11.0 ppb during the 16:00–20:00 time slot, marking the highest value of the entire observation period. Σ24OVOCs began to decline gradually from 29 October. During the December observation period, Σ24OVOCs concentrations generally showed a stable or slightly decreasing trend, with overall concentration levels being relatively low.

Table 2 presents the average concentrations of the 24 carbonyl compounds observed at the Shangdianzi background station during autumn and winter 2024. The average mixing ratio of Σ24OVOCs was 2.70 ± 1.55 ppb. The average concentrations of formaldehyde, acetone, and acetaldehyde were 2.10 ± 1.15 ppb, 0.27 ± 0.29 ppb, and 0.19 ± 0.19 ppb, respectively. Their contribution rates were 77.71%, 9.93%, and 6.89%, collectively accounting for 94.53% of the total Σ24OVOCs concentration. This clearly demonstrates the dominant role of low-carbon OVOCs in the carbonyl composition at this background site, a result consistent with studies reported from multiple urban areas in China [42,49,50,51,52]. It is noteworthy that this study detected various higher-carbon-number carbonyl compounds (≥C5) at the background site, with an average contribution of 3.50%. Although their proportion is not high, their relatively strong photochemical reactivity suggests their potential role in processes leading to enhanced regional oxidation cannot be overlooked [53].

Table 3 compares the concentration levels of atmospheric carbonyl compounds observed in this study with those reported for other cities in China. It should be noted that the literature data used for comparison are primarily derived from observations made in spring and summer, whereas the data in this study were collected during autumn and winter. It is well established that carbonyl compound concentrations typically exhibit a seasonal pattern of being higher in summer and lower in winter. Therefore, directly comparing the autumn-winter background values from this study with spring-summer observations from other sites may overestimate the concentration difference between the background station and urban stations. As an atmospheric environmental background monitoring station, the Shangdianzi site exhibits lower carbonyl compound concentration levels compared to most urban monitoring sites, and its levels are comparable to those observed at typical rural sites or high-altitude/plateau background stations. The average formaldehyde concentration at the Shangdianzi station was only higher than that reported at the Xinglongshan mountain site in 2018 [54] (1.29 ± 1.02 ppb) and the Fuzhou site in 2018 [55] (1.64 ± 0.75 ppb). It was essentially similar to values reported for the plateau city Lhasa in 2022 [56] (2.15 ± 1.72 ppb) and the Nanjing site in 2017 [57] (2.13 ± 0.87 ppb). In the present study, apart from formaldehyde, the concentrations of other carbonyl compounds were all lower than those reported at other sites in the literature. These observed values reflect the representativeness of Shangdianzi as a regional background station and indicate that the area is less directly influenced by local anthropogenic emissions. However, it may be impacted by cross-regional transport and photochemical secondary formation processes, which will be discussed in detail in Section 2.4.

Observations of higher-carbon aldehydes in other regions of China have frequently shown elevated concentrations for 2,5-dimethylbenzaldehyde, n-Valeraldehyde, and n-nonanal. For instance, a typical urban site in Beijing in 2021 detected a relatively high concentration of nonanal at 0.68 ppb [39]. Observations conducted in seven urban and two rural areas in China during 2010 and 2011 also reported elevated levels of nonanal [13]. In a rural area of Dongying City, concentrations of Valeraldehyde and 2,5-dimethylbenzaldehyde were observed at 1.89 ppb and 0.46 ppb, respectively [58]. In the present study, the average concentrations of 2,5-dimethylbenzaldehyde, n-Valeraldehyde, and n-nonanal, as the primary higher-carbon aldehydes, were (43 ± 34) × 10^−3^ ppb, (13 ± 15) × 10^−3^ ppb, and (12 ± 6) × 10^−3^ ppb, respectively, collectively contributing 2.51% to the total Σ24OVOCs. Higher-carbon aldehydes play a significant role in processes such as SOA formation and radical cycling [53]. Their long-term impact on atmospheric oxidative capacity in background regions warrants further attention and investigation.

**Table 3:** Comparison of major carbonyl compound concentrations between the Shangdianzi station and other cities in China (unit: ppb).

City	Time	Site Type	Major Carbonyl Compounds				Reference
			Formaldehyde	Acetaldehyde	Acetone	Propanal	
Shangdianzi	Oct–Nov 2024	Background Site	2.83 ± 1.17	0.26 ± 0.23	0.40 ± 0.36	0.04 ± 0.03	This study
	Dec 2024		1.41 ± 0.58	0.12 ± 0.06	0.14 ± 0.05	0.02 ± 0.01	
Lhasa	Jul 2022	Plateau City	2.15 ± 1.72	0.80 ± 0.72	1.00 ± 0.53	0.09 ± 0.06	[56]
Linyi	Jun 2020	City	3.90 ± 3.60	1.66 ± 1.00	2.03 ± 0.84	0.34 ± 0.16	[50]
Jinan	Jan 2021	City	3.68 ± 1.47	2.83 ± 1.12	3.17 ± 1.41	0.34 ± 0.13	[49]
Huashan	Aug 2020	Mountain Summit	3.40 ± 1.29	1.14 ± 0.52	4.19 ± 1.01	0.08 ± 0.04	[59]
		Mountain Foot	5.40 ± 2.26	1.67 ± 0.69	3.03 ± 0.95	0.13 ± 0.06	
Chengdu	Aug 2019	City	9.86 ± 4.41	3.57 ± 2.19	4.41 ± 2.32	0.38 ± 0.27	[43]
Beijing	May–Sep 2019	City	7.87 ± 3.16	3.17 ± 1.64	5.19 ± 1.84	0.51 ± 0.18	[52]
	Jun–Sep 2020		6.16 ± 3.02	1.67 ± 0.67	2.99 ± 1.20	0.18 ± 0.11	
Beijing	Summer 2015	City	6.90 ± 2.93	2.57 ± 1.20	4.61 ± 1.73	0.29 ± 0.10	[60]
	Winter 2017		3.18 ± 2.40	2.50 ± 2.06	2.57 ± 1.62	0.29 ± 0.19	
	Summer 2018		8.49 ± 2.11	2.97 ± 0.79	6.72 ± 1.58	0.18 ± 0.05	
Zhengzhou	Oct 2018	City	5.22 ± 1.56	3.21 ± 0.87	4.52 ± 1.25	0.55 ± 0.15	[61]
	Jan 2019		4.87 ± 2.56	4.13 ± 2.01	3.80 ± 2.12	0.61 ± 0.23	
	Apr 2019		7.29 ± 1.58	4.94 ± 1.18	4.09 ± 0.75	0.51 ± 0.17	
	Jul 2019		8.34 ± 4.06	5.03 ± 3.27	4.99 ± 1.77	0.47 ± 0.25	
Shijiazhuang	2018	City	3.76 ± 2.29	2.65 ± 1.74	6.46 ± 5.25	0.43 ± 0.42	[54]
Xinglong	2018	Mountain Area	1.29 ± 1.02	0.72 ± 0.48	1.85 ± 1.27	0.11 ± 0.15	[54]
Shantou	2018	City	4.12 ± 1.02	1.57 ± 0.30	7.55 ± 2.10	0.12 ± 0.03	[62]
Nanjing	Mar 2017	City	2.13 ± 0.87	1.25 ± 0.65	1.07 ± 0.62	0.19 ± 0.11	[57]
Fuzhou	May 2018	City	1.64 ± 0.75	4.84 ± 3.63	6.82 ± 8.11	0.54 ± 0.51	[55]
		Suburban Area	2.54 ± 2.09	4.41 ± 4.36	7.45 ± 8.13	0.75 ± 0.82	

#### 3.2.2. Analysis of Seasonal Variations in Carbonyl Compound Concentrations

**Figure 4:**
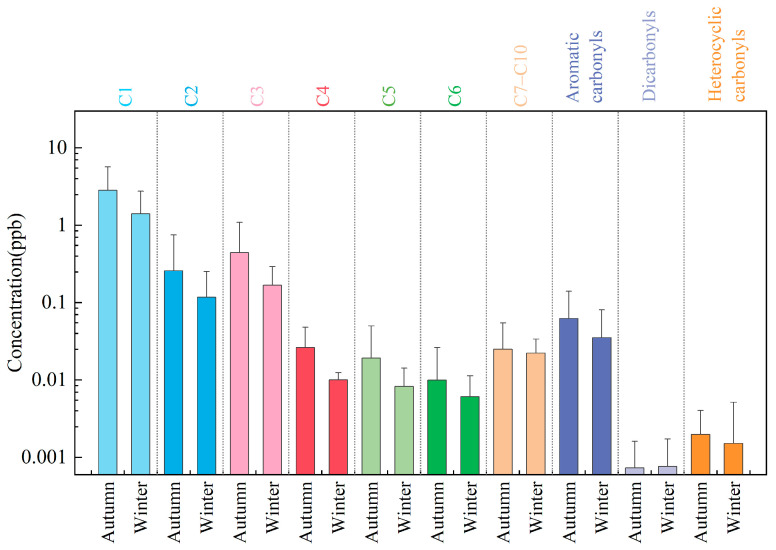
Concentrations of atmospheric carbonyl compounds during autumn and winter 2024 at the Shangdianzi station. (C1: Formaldehyde; C2: Acetaldehyde; C3: Acetone + Propanal + Acrolein; C4: Crotonaldehyde + Methacrolein + 2-Butanone; C5: n-Valeraldehyde + Isovaleraldehyde; C6: n-Hexanal + Cyclohexanone + Methyl isobutyl ketone; C7–C10: n-Heptanal + n-Octanal + n-Nonanal + n-Decanal; Heterocyclic Carbonyls: 2-Furaldehyde; Dicarbonyls: Glutaraldehyde; Aromatic Carbonyls: Benzaldehyde + o-Tolualdehyde + m-Tolualdehyde + p-Tolualdehyde + 2,5-Dimethylbenzaldehyde).

The average concentrations of various carbonyl compounds in the atmosphere during autumn and winter 2024 at the Shangdianzi station are shown in Figure 4, revealing significant seasonal differences. The average concentration in autumn was 3.68 ± 1.66 ppb, while in winter it was 1.78 ± 0.58 ppb, approximately 2.1 times higher in autumn. This distinct “higher in autumn, lower in winter” pattern aligns with the seasonal characteristics reported for urban sites in cities such as Zhengzhou [61], Changsha [63], and Beijing [24,64]. A 2014 study in Foshan [65] reported the highest carbonyl compound concentrations in winter, which is likely related to Foshan’s location in southern China and its subtropical monsoon climate.

With the exception of glutaraldehyde, n-octanal, and n-decanal, the concentrations of all other 23 carbonyl compounds showed a trend of autumn > winter. Among the major species, the decreases in formaldehyde, acetone, and acetaldehyde from autumn to winter were particularly pronounced, with reduction rates of 50.1%, 64.3%, and 54.4%, respectively. The highest decrease rate was observed for isovaleraldehyde, reaching 84.1%. Overall, the seasonal reduction rate for Σ24OVOCs was 51.6%, with the total concentration in winter being approximately half of that in autumn.

**Figure 5:**
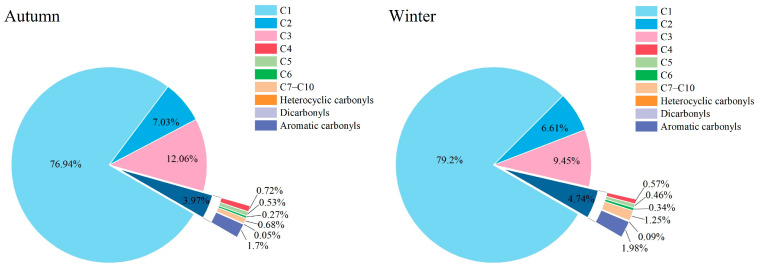
Average percentage contributions of different carbonyl compound groups during autumn and winter at the Shangdianzi station. (C1: Formaldehyde; C2: Acetaldehyde; C3: Acetone + Propanal + Acrolein; C4: Crotonaldehyde + Methacrolein + 2-Butanone; C5: n-Valeraldehyde + Isovaleraldehyde; C6: n-Hexanal + Cyclohexanone + Methyl isobutyl ketone; C7–C10: n-Heptanal + n-Octanal + n-Nonanal + n-Decanal; Heterocyclic Carbonyls: 2-Furaldehyde; Dicarbonyls: Glutaraldehyde; Aromatic Carbonyls: Benzaldehyde + o-Tolualdehyde + m-Tolualdehyde + p-Tolualdehyde + 2,5-Dimethylbenzaldehyde.).

The seasonal differences in the contribution percentages of individual species further reflect their source characteristics. As shown in Figure 4 and Figure 5, the top three compounds in both autumn and winter were formaldehyde, acetone, and acetaldehyde. Their concentration contributions in autumn were 76.9%, 10.9%, and 7.0%, respectively. In winter, the contributions were 79.2%, 8.0%, and 6.6%. This represents a 2.3% increase in formaldehyde’s contribution, while the contributions of acetone and acetaldehyde decreased by 2.9% and 0.4%, respectively. Detailed source apportionment will be discussed in Section 3.4.

#### 3.2.3. Analysis of Diurnal Variations in Carbonyl Compound Concentrations

**Figure 6:**
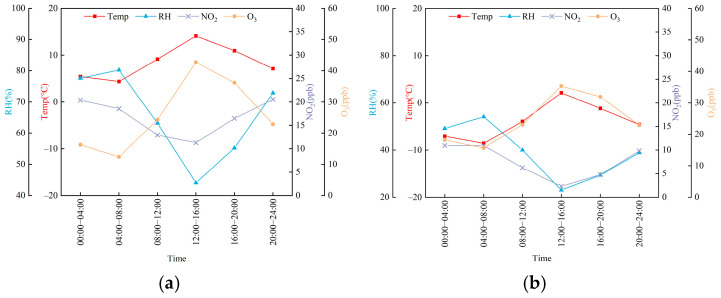
(**a**) Diurnal variations in temperature, relative humidity, NO_2_, and O_3_ in autumn; (**b**) Diurnal variations in temperature, relative humidity, NO_2_, and O_3_ in winter.

**Figure 7:**
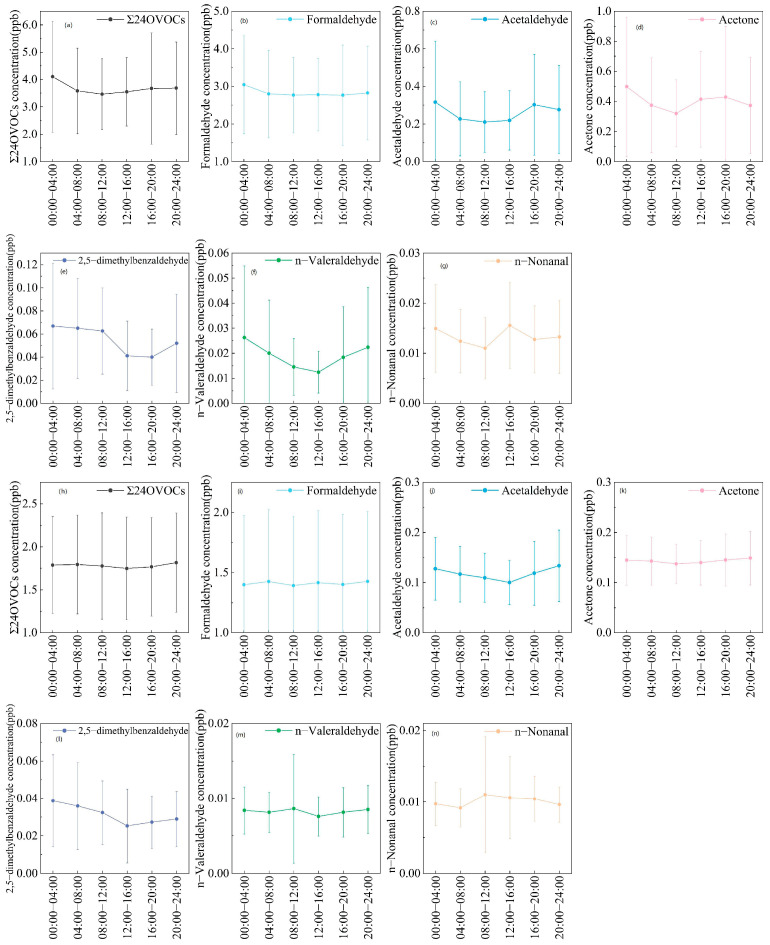
(**a**) Diurnal variation in Σ24OVOCs concentration in autumn; (**b**–**d**) Diurnal variations in the three carbonyl compounds with the highest concentrations (formaldehyde, acetaldehyde, acetone) in autumn; (**e**–**g**) Diurnal variations in the three higher-carbon carbonyl compounds with the highest concentrations (2,5-dimethylbenzaldehyde, n-valeraldehyde, n-nonanal) in autumn; (**h**) Diurnal variation in Σ24OVOCs concentration in winter; (**i**–**k**) Diurnal variations in the three carbonyl compounds with the highest concentrations (formaldehyde, acetaldehyde, acetone) in winter; (**l**–**n**) Diurnal variations in the three higher-carbon carbonyl compounds with the highest concentrations (2,5-dimethylbenzaldehyde, n-valeraldehyde, n-nonanal) in winter.

Figure 6 presents the diurnal variations in temperature, humidity, NO_2_, and O_3_ from autumn 2024 to winter 2025 at Shangdianzi, while Figure 7 shows the diurnal variations in Σ24OVOCs concentration, the concentrations of the top three carbonyl compounds, and the top three higher-carbon carbonyl compounds during the same period. During the autumn observations, three of the six carbonyl compounds—formaldehyde, 2,5-dimethylbenzaldehyde, and n-Valeraldehyde exhibited a consistent diurnal pattern characterized by “higher concentrations at night and lower concentrations during the day”: concentrations peaked during the 00:00–04:00 period, gradually declined thereafter, reached their lowest point around 14:00, and increased again in the evening. This pattern was broadly similar to the diurnal trends of humidity and NO_2_ but opposite to those of temperature and O_3_. Acetaldehyde and acetone showed a gradual decrease from 00:00 to 12:00, but an increase was observed from 12:00 to 20:00, with peaks occurring between 16:00 and 20:00. In contrast to acetaldehyde and acetone, n-nonanal exhibited a peak during the 12:00–16:00 period. The diurnal patterns in winter were similar yet distinct from those in autumn. Formaldehyde concentrations fluctuated, with peaks appearing during 04:00–08:00, 12:00–16:00, and 20:00–24:00. Acetaldehyde, acetone, and 2,5-dimethylbenzaldehyde all showed a trend of initial decrease followed by an increase, with nighttime concentrations generally higher than daytime levels. However, the timing of the lowest concentrations differed: the minima for acetaldehyde and 2,5-dimethylbenzaldehyde occurred during 12:00–16:00, while for acetone it was during 08:00–12:00. n-Valeraldehyde exhibited a peak during 08:00–12:00, and n-nonanal reached its highest concentration during 08:00–12:00 before declining. Unlike the other five carbonyl compounds, n-nonanal showed higher concentrations during the daytime than at night. Previous studies on carbonyl compounds in urban Beijing have also observed high concentrations of n-nonanal [39]. The elevated daytime levels of n-nonanal at Shangdianzi are likely attributable to regional transport rather than being solely controlled by local emissions and photochemical processes.

The “higher at night, lower during the day” characteristic of carbonyl compounds is likely influenced by atmospheric chemical processes [1,2]. During the daytime, carbonyl compounds are primarily consumed through rapid reactions with OH radicals and photolysis. At night, the reaction rate with NO_3_ radicals is much slower than that with OH radicals, allowing most carbonyl species to accumulate [66]. Additionally, the diurnal variation in the atmospheric boundary layer height plays a significant role: solar radiation during the day causes the boundary layer to rise, diluting pollutants, while at night, the lowering of the boundary layer facilitates the accumulation of carbonyls and their precursors [67,68,69]. It is noteworthy that for background regions, the influence of regional transport on carbonyl concentrations cannot be overlooked. Unusually high concentrations of some carbonyl compounds during specific periods may indicate the importance of regional transport. In future studies, combining backward trajectory analysis with chemical transport models is expected to more accurately quantify the role of regional transport. Overall, the diurnal variation of carbonyl compounds at the Shangdianzi station reflects the combined effects of photochemical reactions, regional transport, and boundary layer height. The source apportionment will be discussed in detail in Section 3.4.

### 3.3. Analysis of Atmospheric Photochemical Reactivity of Carbonyl Compounds

**Figure 8:**
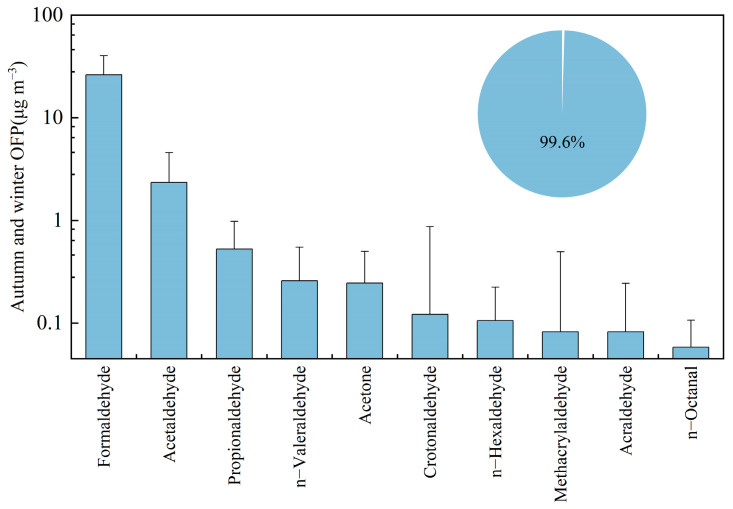
Top ten atmospheric carbonyl compounds ranked by OFP and their combined contribution to the total OFP during the observation period at Shangdianzi.

To quantitatively assess the contribution of carbonyl compounds to ozone formation, this study calculated OFP of carbonyls at the Shangdianzi station during autumn and winter using MIR method. The average OFP of Σ24OVOCs during the observation period was 30 ± 16 μg/m^3^ (Note: 2-furaldehyde, 2,5-dimethylbenzaldehyde, n-nonanal, and n-decanal were excluded from the OFP calculation due to lack of MIR values). Figure 8 summarizes the top ten carbonyl species ranked by the OFP, whose combined OFP accounted for 99.6% of the total Σ24OVOCs OFP, demonstrating their dominant role. Formaldehyde, acetaldehyde, and propanal were the top three contributors, with OFP values of 26 ± 14 μg/m^3^, 2 ± 2 μg/m^3^, and 0.5 ± 0.4 μg/m^3^, respectively. Their combined contribution accounted for 96.5% of the total Σ24OVOCs OFP.

At the Shangdianzi station, formaldehyde contributes the most to O_3_ formation due to its high concentration and photochemical reactivity, accounting for 86.9% of the total OFP. This contribution rate is generally higher than observations from urban sites such as Beijing [24] (66.5%), Shenzhen [70] (39%), and Shijiazhuang [54] (64.0%), highlighting the dominance of formaldehyde in photochemistry within this background area. Acetaldehyde has an OFP contribution of 7.8%, ranking second. Although acetone has a relatively high concentration, its contribution to ozone formation is minor due to its low photochemical reactivity, with an OFP contribution of only 0.8%. Among the higher-carbon carbonyls, n-Valeraldehyde, n-hexanal, and n-octanal have the highest OFP values at 0.3 ± 0.3 μg/m^3^, 0.1 ± 0.1 μg/m^3^, and 0.1 ± 0.1 μg/m^3^, respectively. However, considering their potential for SOA formation, their environmental significance should not be overlooked [66]. It is important to note that the MIR values for aromatic carbonyl compounds (benzaldehyde, o-/m-/p-tolualdehyde) in this study were negative, resulting in negative calculated OFP values. This indicates that these aromatic carbonyls have a net consumption effect on ozone during atmospheric photochemical reactions. This characteristic suggests that aromatic carbonyls in the background area may be more involved in HO_2_ and RO_2_ radical cycling rather than directly promoting ozone generation [1,41,71].

**Figure 9:**
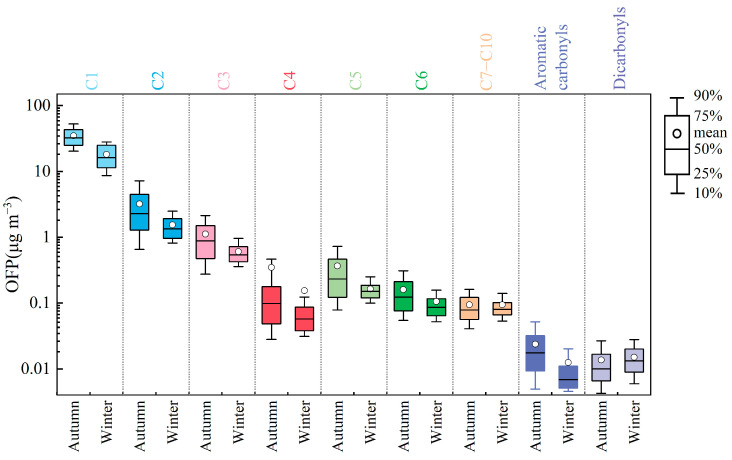
OFP of atmospheric carbonyl compounds during autumn and winter at the Shangdianzi station. (Note: The OFP values for aromatic carbonyl compounds are negative. Their absolute values are displayed in the figure and are distinguished by a borderless format. C1: Formaldehyde; C2: Acetaldehyde; C3: Acetone + Propanal + Acrolein; C4: Crotonaldehyde + Methacrolein + 2-Butanone; C5: n-Valeraldehyde + Isovaleraldehyde; C6: n-Hexanal + Cyclohexanone + Methyl isobutyl ketone; C7–C10: n-Heptanal + n-Octanal + n-Nonanal + n-Decanal; Heterocyclic Carbonyls: 2-Furaldehyde; Dicarbonyls: Glutaraldehyde; Aromatic Carbonyls: Benzaldehyde + o-Tolualdehyde + m-Tolualdehyde + p-Tolualdehyde + 2,5-Dimethylbenzaldehyde).

The OFP of carbonyl compounds at Shangdianzi during autumn and winter is shown in Figure 9. The average OFP values for Σ24OVOCs were 40 ± 17 μg/m^3^ in autumn and 21 ± 7 μg/m^3^ in winter. The autumn OFP was significantly higher than the winter value, approximately 1.93 times that of winter, consistent with the seasonal concentration pattern. With the exception of glutaraldehyde and n-octanal, the OFP values of all other carbonyl compounds followed the trend autumn > winter. In both seasons, the top three species by OFP were formaldehyde, acetaldehyde, and propanal. Their respective contributions in autumn were 86.8%, 8.0%, and 1.7%, while in winter they were 87.2%, 7.4%, and 1.9%. Although the seasonal variation in the percentage contributions was minor, formaldehyde consistently maintained an absolute dominant position, underscoring its critical role in the regional background atmosphere.

Overall, the contribution of carbonyl compounds to ozone formation at the Shangdianzi station exhibits clear species-specific characteristics. Formaldehyde is the most crucial precursor, demonstrating extremely high photochemical reactivity even in a background region. Controlling high-reactivity species such as formaldehyde holds significant importance in background areas. While higher-carbon aldehydes made limited contributions to OFP in this study, their potential role in SOA formation warrants further attention.

### 3.4. Source Apportionment of Carbonyl Compounds

Carbonyl compounds enter the atmosphere either through direct primary emissions from sources such as vehicles, industrial processes, and combustion, or via secondary formation through the photochemical oxidation of VOCs. Their sources are typically complex and region-specific [13,66]. To more comprehensively identify the source characteristics of carbonyl compounds during autumn and winter at the Shangdianzi background station, this study employed an integrated approach utilizing characteristic ratios, correlation analysis, and multiple linear regression for source apportionment [72,73,74]. The findings are further discussed in conjunction with the seasonal and diurnal variation characteristics described previously.

#### 3.4.1. Characteristic Ratios

The concentration ratios of formaldehyde/acetaldehyde (C1/C2) and acetaldehyde/propanal (C2/C3) are often used as important indicators for identifying potential sources of carbonyl compounds. Generally, VOCs emitted from natural sources (e.g., isoprene, monoterpenes) tend to generate more formaldehyde than acetaldehyde during atmospheric oxidation. Therefore, C1/C2 ratios are typically higher in areas dominated by vegetation emissions [72]. In contrast, in urban areas dominated by anthropogenic emissions (e.g., vehicle exhaust, solvent use, industrial emissions), the relative proportion of acetaldehyde increases, leading to a significant decrease in the C1/C2 ratio [59]. Previous studies have shown that the C1/C2 concentration ratio typically ranges between 1 and 2 in urban areas significantly influenced by human activities, while in forested and remote areas, it can reach approximately 10 [72]. Similarly, propanal primarily originates from anthropogenic sources such as industrial and vehicular emissions. Consequently, the C2/C3 concentration ratio is usually lower in urban areas and relatively higher in regions where natural sources dominate [27].

**Figure 10:**
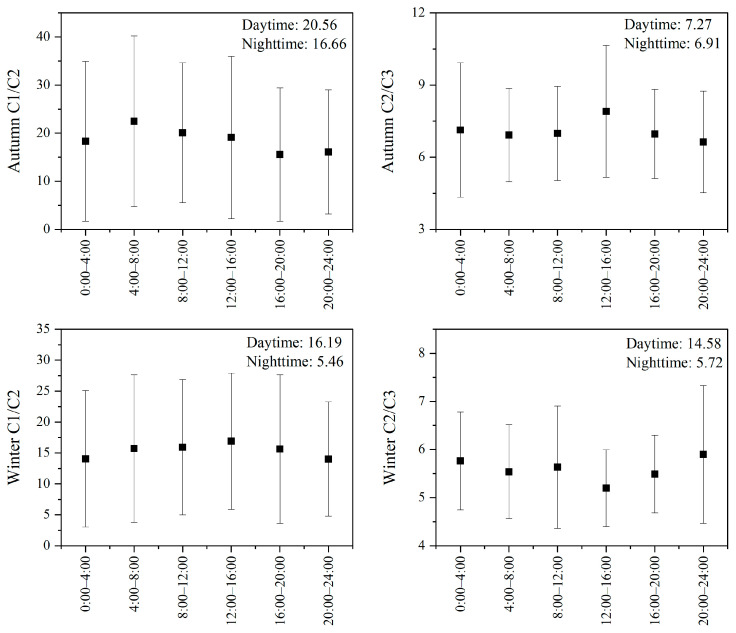
C1/C2 and C2/C3 ratios during autumn and winter at the Shangdianzi background station.

As shown in Figure 10, the average atmospheric C1/C2 ratios at the Shangdianzi station were 18.6 (range:2.52–73.98) in autumn and 15.3 (range: 3.08–62.31) in winter, while the C2/C3 ratios were 7.2 (range: 2.34–14.69) and 5.6 (range: 3.74–7.99), respectively. Both C1/C2 and C2/C3 ratios were higher in autumn than in winter, indicating a greater contribution from natural sources and a smaller contribution from anthropogenic sources to carbonyl compounds in autumn compared to winter. The C1/C2 ratios observed in this study were significantly higher than those reported for urban and suburban areas in Beijing and other cities across northern and southern China during autumn and winter [25,47,75]. The C2/C3 ratios were lower than those reported for urban Beijing in autumn and winter (8.3) [24] and Foshan (14.35) [65], higher than that in Hong Kong (4) [76], and similar to that in Taiyuan (5.1) [25]. This comparative analysis indicates that the Shangdianzi station, as a regional background site, exhibits source characteristics for carbonyls that are in a state of “dominated by natural sources with superimposed anthropogenic influences.”

From the perspective of diurnal variation, the C1/C2 ratio in both autumn and winter exhibited a pattern of being higher during the day and lower at night. The higher daytime C1/C2 values can be attributed to two main factors: firstly, daytime plant emissions and the secondary formation of formaldehyde from VOCs; secondly, the photochemically driven production rate of formaldehyde is generally higher than that of acetaldehyde [77]. The lower nighttime C1/C2 ratios are likely related to reduced biogenic emissions at night, while acetaldehyde continues to receive contributions from anthropogenic sources such as combustion. In autumn, the C2/C3 ratio showed a distinct peak during the 12:00–16:00 period. This may be associated with a certain degree of secondary formation of acetaldehyde (C2) during the day, combined with the fact that propanal has a higher reaction rate with OH radicals than formaldehyde, leading to its increased consumption during this period [27]. In winter, the C2/C3 ratio displayed a pattern of being lower during the day and higher at night. This suggests that under the conditions of weakened photochemical activity and reduced biogenic emissions in winter, anthropogenic emissions of propanal are relatively more stable, making it more prone to nighttime accumulation compared to acetaldehyde [74].

Integrating the findings from the characteristic ratios and their diurnal variation patterns, it can be concluded that atmospheric carbonyl compounds at the Shangdianzi station in autumn primarily originate from natural sources and secondary formation, with a relatively minor influence from anthropogenic sources. In winter, against a backdrop of significantly weakened natural source contributions, the relative importance of anthropogenic sources substantially increases, resulting in a composite pattern where both anthropogenic and natural sources jointly influence carbonyl levels.

#### 3.4.2. Correlation Analysis

**Figure 11:**
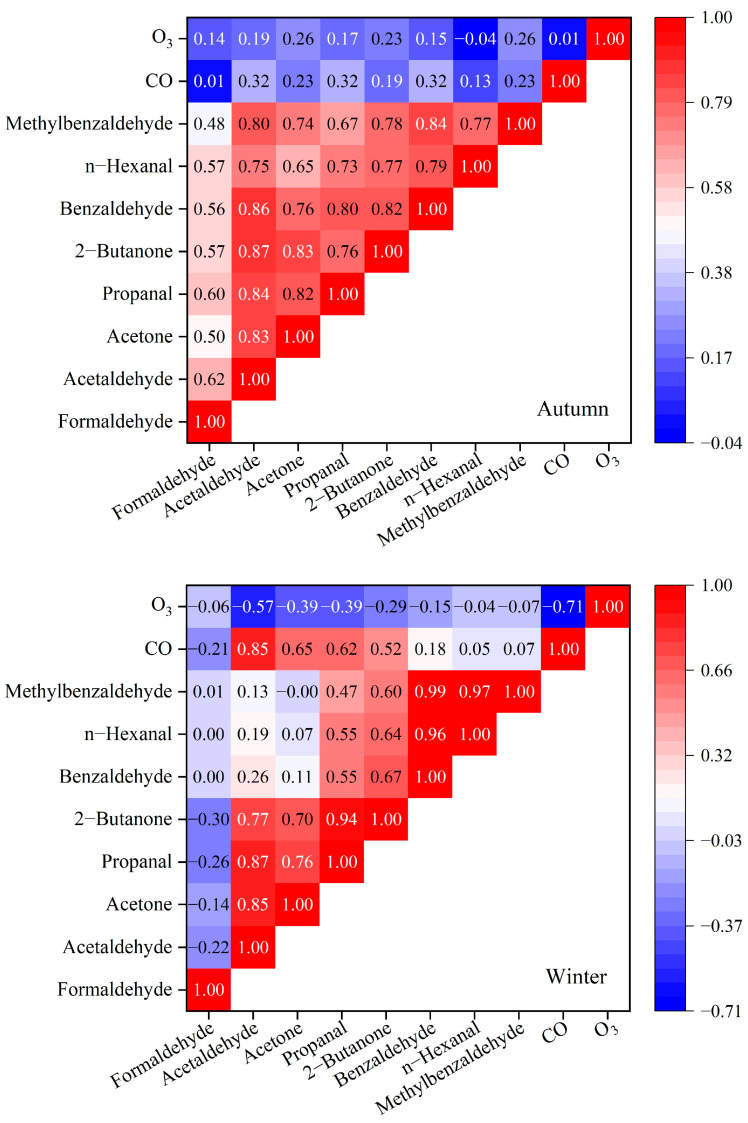
Correlation matrix of atmospheric carbonyl compounds, O_3_, and CO during autumn and winter at the Shangdianzi background station.

Atmospheric carbonyl compounds originate from both primary emissions and secondary formation via photochemical reactions. Pollutants with similar sources often exhibit certain correlations [73]. To further analyze the source relationships of carbonyl compounds in the Miyun District atmosphere of Beijing, Pearson correlation analysis was performed on the observed concentrations of carbonyl compounds at the Shangdianzi station for autumn and winter, respectively. The results are shown in Figure 11. n-Hexanal and propanal are considered to originate mainly from emissions of chemical enterprises [78,79]; benzaldehyde and methylbenzaldehydes primarily come from gasoline vehicle exhaust [78,80]; 2-butanone mainly originates from industrial emissions and solvent use [81]; O_3_ and CO were used as tracers for secondary formation and primary sources (fuel combustion and vehicle exhaust), respectively [44].

In autumn, all carbonyl compounds showed relatively moderate or strong positive correlations (0.48–0.87). Formaldehyde had relatively weaker correlations with other carbonyls (0.48–0.62), while correlations among carbonyls other than formaldehyde were stronger (0.65–0.87). All carbonyls exhibited weak correlations with O_3_ and CO, with the correlation coefficient between formaldehyde and CO being close to zero (0.01). This suggests that formaldehyde in autumn primarily originated from natural sources but was also influenced to some extent by anthropogenic sources (vehicles and industry) and secondary formation. Other carbonyl compounds mainly originated from anthropogenic sources (vehicle exhaust and industrial emissions), with some species also influenced by secondary production.

In winter, acetaldehyde, acetone, propanal, and 2-butanone showed strong positive correlations (0.70–0.94). Propanal, 2-butanone, benzaldehyde, n-hexanal, and methylbenzaldehyde also exhibited moderate or strong positive correlations (0.47–0.99), reflecting that these species likely share similar or identical primary emission sources. Acetaldehyde, acetone, propanal, and 2-butanone had moderate and strong positive correlations with CO (0.52–0.85) and moderate negative correlations with O_3_ (0.39–0.57). Formaldehyde showed some correlation with acetaldehyde, propanal, 2-butanone, and CO (0.22–0.30). This indicates that formaldehyde in winter primarily originated from natural sources but was influenced to some extent by anthropogenic sources (combustion and industrial emissions). Other carbonyl compounds were mainly derived from anthropogenic sources: acetaldehyde and acetone primarily from combustion and industrial emissions; propanal and 2-butanone mainly from combustion, vehicle, and industrial emissions; and benzaldehyde, n-hexanal, and methylbenzaldehydes primarily from vehicle exhaust and industrial sources.

#### 3.4.3. Multiple Linear Regression

**Figure 12:**
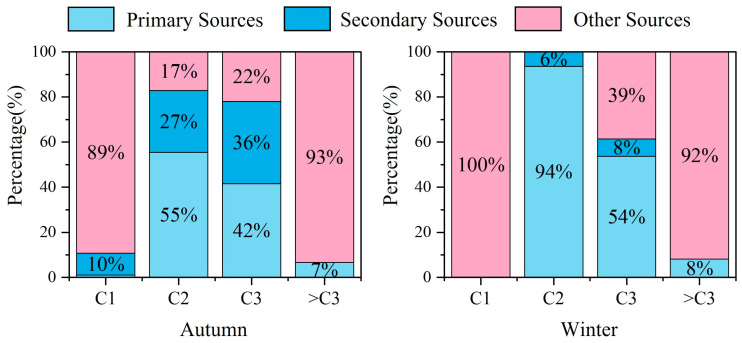
Relative contributions of primary, secondary, and other sources to atmospheric carbonyl compounds during autumn and winter at the Shangdianzi background station (C1 represents formaldehyde, C2 represents acetaldehyde, and C3 represents acetone + propanal + acrolein.).

Building upon the characteristic ratios and correlation analyses, to further quantify the contributions of primary sources, secondary sources, and other sources to carbonyl compounds, CO and O_3_ were used as tracers for primary and secondary sources, respectively. Multiple linear regression analysis was employed to apportion the sources of carbonyl compounds at the Shangdianzi station in Beijing. The regression coefficients are presented in Table 4, and the relative contributions of the various sources are shown in Figure 12.

The use of CO and O_3_ as tracers proved inadequate for the source apportionment of C1 in both autumn and winter, as indicated by *p* > 0.05 and negative values for β_1_ and β_2_. However, this source apportionment lacks specific tracers related to biogenic (plant) emissions [82]. This is likely because formaldehyde primarily originates from natural sources. In contrast, the source apportionment results for C2 and propanal C3 were reliable (*p* < 0.01). The analysis revealed that primary sources were the dominant contributors to both C2 and C3 (42–94%). Additionally, in autumn, C2 and C3 showed significant contributions from secondary sources (27–36%) and other anthropogenic sources (17–22%). In winter, C3 also contained a notable proportion from other anthropogenic sources (39%). The source apportionment results for species with carbon numbers >C3 were less satisfactory. Previous studies have found that the explanatory power of multiple linear regression decreases with increasing carbon number of carbonyl compounds, as higher-carbon carbonyls may have more complex sources [68]. Based on the combined results of the ratio method and correlation analysis, a further analysis of the specific sources of carbonyl compounds reveals the following: In both autumn and winter, the “other sources” of C1 are primarily natural sources. In autumn, the “primary sources” and “other sources” of C2, C3, and >C3 species mainly include anthropogenic emissions such as vehicle exhaust and industrial releases. In winter, the “primary sources” and “other sources” of C2 and C3 are predominantly anthropogenic, including combustion sources, vehicle emissions, and industrial releases, while the “other sources” of >C3 species mainly originate from vehicle exhaust and industrial emissions.

Integrating the findings from the correlation analysis above, it can be concluded that carbonyl compounds at the Shangdianzi station in autumn are characterized by the combined influence of natural sources, secondary formation, and anthropogenic sources such as vehicle exhaust and industrial emissions. Specifically, C1 is dominated by natural sources and secondary formation, while C2 and C3 exhibit characteristics of both primary and secondary sources. In winter, the contributions from natural sources and photochemical secondary formation decrease significantly. In contrast, the contributions from primary sources and other anthropogenic sources to C2 and C3 increase markedly. Factors such as combustion sources, heating emissions, vehicle exhaust, and industrial releases become the primary controlling factors. The sources of higher-carbon carbonyl species are more diverse. Beyond local anthropogenic emissions, they may also be significantly influenced by regional transport and heterogeneous reactions. Future research should combine trajectory analysis and source apportionment models to further investigate these aspects.

## 4. Conclusions

The average concentration of Σ24OVOCs in the atmosphere at the Shangdianzi station during autumn and winter was 2.70 ± 1.55 ppb, with formaldehyde, acetone, and acetaldehyde being the predominant components, collectively accounting for 94.5% of the total. Formaldehyde, acetaldehyde, and propanal were the major contributors to OFP, with an average OFP value of 30 ± 16 μg/m^3^. Formaldehyde alone contributed 86.9%, indicating it is the most critical carbonyl species for ozone formation in this background region. Compared with urban sites in China, both the carbonyl concentrations and OFP at the Shangdianzi station were generally at lower levels, reflecting the regional environmental characteristics of a background site.

During the observation period, carbonyl compounds exhibited a significant seasonal variation characterized by higher concentrations in autumn than in winter. The average Σ24OVOCs concentration in autumn (3.68 ± 1.66 ppb) was approximately 2.1 times that in winter (1.78 ± 0.58 ppb). Diurnally, most carbonyl species showed a pattern of higher concentrations at night and lower concentrations during the day, with peaks occurring in the 00:00–04:00 period. This pattern was similar to the variation in NO_2_ but opposite to the diurnal trends of O_3_ and temperature. Variations in carbonyl compound concentrations are influenced by a combination of photochemical reactions, regional transport, and changes in boundary layer height.

Atmospheric carbonyl compounds at the Shangdianzi station in autumn were influenced by a combination of natural, anthropogenic, and secondary sources. Specifically, C1 (formaldehyde) primarily originated from natural sources. C2 (acetaldehyde) and C3 (primarily acetone and propanal), along with other carbonyl compounds, are mainly derived from anthropogenic sources such as vehicle exhaust and industrial emissions (64–100%). Secondary sources also contributed to C2 and C3 (27–36%). In winter, atmospheric carbonyl compounds were jointly influenced by natural and anthropogenic sources. C1 (formaldehyde) primarily came from natural sources, while C2 (acetaldehyde) and C3 (primarily acetone and propanal) mainly originated from anthropogenic sources such as combustion, vehicle, and industrial emissions (93–94%). Species with carbon numbers >C3 (other carbonyl compounds) were almost exclusively (100%) attributed to anthropogenic sources, specifically vehicle exhaust and industrial emissions.

## Figures and Tables

**Table 1:** Information, MS Acquisition Parameters (Q1 represents the quadrupole one, and its unit is mass-to-charge ratio (*m*/*z*)), Method Detection Limit (MDL), and Determination Coefficients (R^2^) for the 24 Carbonyl-DNPH Derivatives.

No.	Compound	Derivative CAS No.	Retention Time (min)	Purity (%)	Molecular Weight (Da)	MIR (gO_3_/ gVOC)	Q1 Mass (*m*/*z*)	MDL (ng/mL)	R^2^
1	Formaldehyde	1081-15-8	5.00	99.7	210.15	9.46	208.9	0.4	0.995
2	Acetaldehyde	1019-57-4	6.24	99.8	224.17	6.54	222.7	0.5	0.992
3	2-Furaldehyde	2074-2-4	7.38	99.7	276.21	--	274.9	0.5	0.998
4	Acrolein	888-54-0	7.78	99.6	236.18	7.45	235.0	0.6	0.999
5	Acetone	1567-89-1	8.09	99.8	238.20	0.36	236.8	0.4	0.997
6	Propanal	725-00-8	8.63	97.3	238.20	7.08	236.8	0.5	0.999
7	Crotonaldehyde	1527-96-4	10.42	99.1	250.21	9.39	249.1	0.5	0.999
8	Methacrolein	5077-73-6	11.16	99.9	250.21	6.01	249.1	0.5	0.999
9	2-Butanone	958-60-1	11.85	99.9	252.23	1.48	251.1	0.2	0.999
10	Benzaldehyde	1157-84-2	13.07	99.9	286.24	−0.67	285.7	0.5	0.999
11	Glutaraldehyde	5085-7-4	15.35	98.5	460.36	4.31	459.1	0.9	0.993
12	Isovaleraldehyde	2256-1-1	15.96	99.9	266.25	4.97	265.0	0.7	0.998
13	Cyclohexanone	1589-62-4	16.08	99.3	278.26	1.35	277.1	0.8	0.996
14	n-Valeraldehyde	2057-84-3	16.84	99.9	266.25	5.08	265.0	0.9	0.999
15	o-Tolualdehyde	1773-44-0	17.86	99.9	300.27	−0.59	298.8	0.5	0.988
16	m-Tolualdehyde	2880-5-9	18.48	99.8	300.27	−0.59	298.8	0.5	0.992
17	p-Tolualdehyde	2571-00-8	19.25	99.9	300.27	−0.59	298.8	0.6	0.995
18	Methyl isobutyl ketone	1655-42-1	21.86	99.8	280.28	3.88	279.1	0.4	0.999
19	n-Hexanal	1527-97-5	23.95	99.9	280.28	4.35	279.1	0.4	0.996
20	2,5-Dimethylbenzaldehyde	152477-96-8	24.41	99.9	314.30	--	313.1	0.4	0.991
21	n-Heptanal	2074-5-7	27.28	98.9	294.31	3.69	293.2	0.3	0.989
22	n-Octanal	1726-77-8	29.10	99.4	308.33	3.16	306.9	0.3	0.988
23	n-Nonanal	2348-19-8	30.40	99.5	322.36	--	321.1	0.2	0.989
24	n-Decanal	1527-95-3	31.40	99.5	336.39	--	335.1	0.4	0.993

**Table 2:** Average concentrations of carbonyl compounds at the Shangdianzi background station during autumn and winter 2024 (unit: ×10^−3^ ppb).

Carbonyl Compound	Autumn	Winter	Average
Formaldehyde	2828 ± 1163	1410 ± 576	2097 ± 1153
Acetaldehyde	258 ± 232	118 ± 59	186 ± 181
2-Furaldehyde	2 ± 5	2 ± 1	2 ± 4
Acrolein	5 ± 11	4 ± 5	4 ± 9
Acetone	401 ± 361	143 ± 48	268 ± 285
Propanal	38 ± 34	21 ± 9	29 ± 26
Crotonaldehyde	5 ± 19	3 ± 30	4 ± 25
Methacrolein	7 ± 27	2 ± 16	4 ± 22
2-Butanone	14 ± 15	5 ± 3	9 ± 12
Benzaldehyde	5 ± 4	3 ± 7	4 ± 6
Glutaraldehyde	1 ± 1	1 ± 0	1 ± 1
Isovaleraldehyde	0 ± 1	0 ± 0	0 ± 1
Cyclohexanone	2 ± 2	1 ± 2	1 ± 2
n-Valeraldehyde	19 ± 20	8 ± 4	13 ± 15
o-Tolualdehyde	2 ± 2	1 ± 1	1 ± 2
m-Tolualdehyde	1 ± 1	0 ± 3	0 ± 2
p-Tolualdehyde	0 ± 1	0 ± 2	0 ± 1
Methyl isobutyl ketone	1 ± 1	1 ± 0	1 ± 1
n-Hexanal	7 ± 5	4 ± 7	6 ± 6
2,5-Dimethylbenzaldehyde	55 ± 41	31 ± 20	43 ± 34
n-Heptanal	2 ± 2	2 ± 1	2 ± 1
n-Octanal	3 ± 2	3 ± 3	3 ± 3
n-Nonanal	13 ± 7	10 ± 5	12 ± 6
n-Decanal	7 ± 3	7 ± 6	7 ± 5
Σ24OVOCs	3676 ± 1660	1781 ± 577	2699 ± 1550

**Table 4:** Multiple linear regression coefficients for atmospheric carbonyl compounds during autumn and winter at the Shangdianzi background station(C1 represents formaldehyde, C2 represents acetaldehyde, and C3 represents acetone + propanal + acrolein.).

	Species	β_0_	β_1_	β_2_ (10^−3^)	*p*
Autumn	C1	2.53	0.09	10.67	>0.05
	C2	0.04	0.44	2.76	<0.01
	C3	0.10	0.57	6.29	<0.01
	>C3	0.14	0.03	−0.08	>0.05
Winter	C1	2.66	−2.85	−20.47	<0.01
	C2	0.00	0.49	0.29	<0.01
	C3	0.06	0.42	0.51	<0.01
	>C3	0.09	0.04	−0.78	>0.05

## Data Availability

The data used in this study are available upon request from the corresponding author.

## Supplementary Materials

The following supporting information can be downloaded at https://www.mdpi.com/article/10.3390/toxics14020156/s1, Figure S1: Wind rose diagram during the observation period at the Shangdianzi station.
