# Supplementary material for: Atmospheric Carbonyl Compounds at Shangdianzi, Beijing: Autumn-to-Winter Variation, Ozone Formation Potential, and Source Apportionment
Source: Toxics. 2026 Feb 4;14(2):156. doi: 10.3390/toxics14020156 (PMC12944414; doi:10.3390/toxics14020156)
Supplement: Supplementary file 1 [file toxics-14-00156-s001.zip › toxics-4102486-supplementary.pdf]

Supplement:

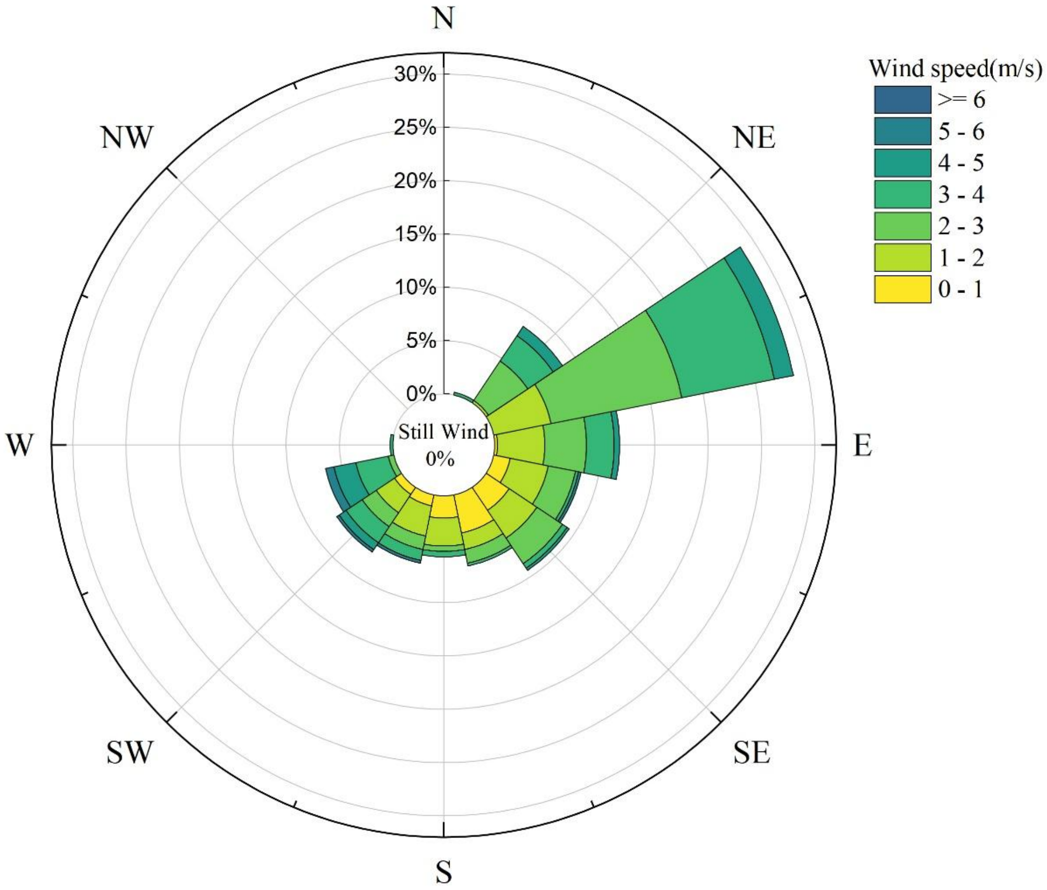

Figure S1. Wind rose diagram during the observation period at the Shangdianzi station.
